# Utility of Liver Biopsy in the Diagnosis and Management of Possible Drug-Induced Liver Injury in Patients Receiving Antituberculosis Therapy: A Retrospective Study

**DOI:** 10.3390/idr15060066

**Published:** 2023-11-28

**Authors:** Gina Gualano, Drieda Zace, Silvia Mosti, Paola Mencarini, Maria Musso, Raffaella Libertone, Carlotta Cerva, Delia Goletti, Alessia Rianda, Franca Del Nonno, Laura Falasca, Fabrizio Palmieri

**Affiliations:** 1 Respiratory Infectious Diseases Unit, National Institute for Infectious Diseases Lazzaro Spallanzani IRCCS, 00149 Rome, Italy; silvia.mosti@inmi.it (S.M.); paola.mencarini@inmi.it (P.M.); maria.musso@inmi.it (M.M.); raffaella.libertone@inmi.it (R.L.); carlotta.cerva@inmi.it (C.C.); delia.goletti@inmi.it (D.G.); alessia.rianda@inmi.it (A.R.); fabrizio.palmieri@inmi.it (F.P.); 2 Clinical Infectious Diseases, Department of System Medicine, University of Rome Tor Vergata, 00133 Rome, Italy; driedazace@gmail.com; 3 Pathology Unit, National Institute for Infectious Diseases Lazzaro Spallanzani IRCCS, 00149 Rome, Italy; franca.delnonno@inmi.it (F.D.N.); laura.falasca@inmi.it (L.F.)

**Keywords:** antitubercular drugs, hepatotoxicity, liver injury, liver biopsy, histology, DILI diagnosis, DILI management

## Abstract

Background: Drug-induced liver injury (DILI) secondary to ATT treatment (TB-DILI) is reported in 2–28% of patients. We present here a series of clinical cases of suspected DILI arising during antituberculosis treatment, studied with the aid of liver biopsy. Methods: this was a retrospective descriptive study including 10 tuberculosis patients who underwent liver biopsy for suspected TB-DILI at the “Lazzaro Spallanzani” Institute from 2017 to 2022. Results: Ten patients who underwent LB were extracted from the database and included in the retrospective study cohort. According to the clinical classification, eight patients had hepatocellular liver injury, one patient had cholestatic injury, and another had mixed-type injury. Histopathological diagnosis revealed liver damage due to DILI in 5/10 (50%) cases. In one case, liver biopsy showed necrotizing granulomatous hepatitis. Conclusions: Severe and persistent elevation of hepatic transaminases, hepatic cholestasis despite discontinuation of therapy, and other suspected hepatic conditions are indications for liver biopsy, which remains a valuable tool in the evaluation of selected tuberculosis patients with suspected DILI for many reasons. However, the decision to perform a liver biopsy should be based on clinical judgment, considering the benefits and risks of the procedure.

## 1. Introduction

Tuberculosis (TB) is a significant public health problem globally, with approximately 10 million cases and 1.4 million deaths per year [1]. Antituberculous therapy (ATT) is the basis of tuberculosis treatment. However, among the common side effects of ATT is hepatotoxicity [2,3]. Drug-induced liver injury secondary to antituberculosis treatment (TB-DILI) is reported in 2–28% of patients, varying according to definition, study sample and treatment regimen. TB-DILI is ultimately a clinical diagnosis of exclusion [4]. Other causes of liver damage, such as acute viral hepatitis, should always be looked for carefully and, in their absence, the diagnosis of DILI appears plausible. In cases of suspected DILI, it is important to assess causality using scoring systems such as the Roussel Uclaf Causality Assessment Method (RUCAM) score [5]. To help define DILI, the DILI Expert Working Group requested consensus on threshold criteria for any drug-related DILI, including criteria based on ALT and ALP [6].

Regarding the management of this condition, the best approach is still a matter of debate. Liver biopsy (LB) is an invasive procedure that can help clinicians in the diagnosis and management of ATT-induced liver injury. In clinical practice, it is performed when the diagnosis is uncertain or there is concern about the severity of the lesion. LB is not mandatory in the DILI assessment and is not required for the RUCAM score. Commonly, the biopsy is performed early and can guide subsequent management such as initiation of steroid therapy or referral for liver transplantation [7]. We conducted a retrospective analysis of a series of patients treated at a tertiary care tuberculosis hospital center in Italy who underwent LB for suspected TB-DILI, with the aim of describing the utility of biopsy for the differential diagnosis of liver disease and its treatment during anti-TB treatment.

## 2. Materials and Methods

We conducted a retrospective descriptive study of a case series including 10 patients who underwent liver biopsy for the diagnosis of TB-DILI at the “Lazzaro Spallanzani” Institute from 2017 to 2022. The patients were treated based on the institutional protocol, which was written following WHO guidelines on tuberculosis. In all cases, an individualized regimen designed based on the results of drug susceptibility tests and adapted to comorbidities was used [8,9,10]. Initial treatment was provided on an inpatient basis, until acid-fast bacillus (AFB) sputum conversion was achieved on three consecutive negative specimens collected over the course of one week. Patients received therapy with direct observation during hospitalization. During hospitalization, laboratory tests were repeated almost weekly, or as needed based on clinical conditions, and monthly or as needed in the subsequent period. Baseline liver tests (LTs) were performed, and all patients were offered baseline screening for HIV and hepatitis B and C. Clinical and blood evaluations were repeated at each visit, including actively and systematically looking for symptoms such as nausea, vomiting, abdominal pain, dark urine, itching and loss of appetite.

Patients with abnormal LTs were managed according to institutional guidelines, which are based on the indications of the ATS [11]. Antituberculous therapy was modified or discontinued in asymptomatic patients with ALT 3-5xULN, who were carefully monitored. In cases of abnormal liver biochemistry, risk factors considered included age, sex, baseline LT, HIV, hepatitis B and C, hepatitis E and A, CMV, EBV, HSV, alcohol consumption and chronic liver disease. ANA, ASMA and LKM-1, anti-neutrophil cytoplasmic AMA (ANCA) and anti-double-stranded DNA were determined in all patients with suspected TB-DILI. Laboratory tests considered included aspartate aminotransferase (AST; normal range 5–40 U/L), alanine aminotransferase (ALT; normal range 5–40 U/L), total bilirubin (T-Bil; normal range 0.2–1.0 mg/dL), alkaline phosphatase (ALP; normal range 40–116 U/L) and gamma-glutamyltransferase (GGT; normal range < 38 U/L). Patients were classified as DILI cases if the following conditions were met: alanine aminotransferase (ALT) levels ≥ 5 times the upper limit of normal, alkaline phosphatase (ALP) ≥ 2 times the upper limit of normal and total bilirubin levels > 2 times the upper limit of normal, after excluding other potential causes.

Classification of DILI patients’ clinical presentation was based on the RUCAM method. The R-ratio value was calculated as follows: ALT value/LT upper limit of the normal range (ULN)/ALP value/ALP ULN. The suspected DILI cases were classified into three patterns: hepatocellular pattern of DILI = R > 5; mixed pattern of DILI = R between 2 and 5; and cholestatic pattern = R < 2 [5].

DILI severity grade was determined as defined by the DILI Expert Working Group, described as follows: Grade 1, mild: elevated alanine aminotransferase/alkaline phosphatase (ALT/ALP) concentration meeting criteria for DILI but bilirubin concentration < 2× the upper limit of normal (ULN). Grade 2, moderate: elevated ALT/ALP concentration meeting criteria for DILI and bilirubin concentration ≥ 2× the ULN, or symptomatic hepatitis. Grade 3, severe: elevated ALT/ALP concentration meeting criteria for DILI, bilirubin concentration ≥ 2× the ULN and one of the following: INR ≥ 1.5; ascites and/or encephalopathy, disease duration < 26 weeks and absence of underlying cirrhosis; and other organ failures considered to be due to DILI. Grade 4, fatal or transplantation: death or transplantation due to DILI [6].

In the event of severe TB, we prescribed a non-hepatotoxic background regimen (according to local protocol) whilst stopping the most hepatotoxic drugs. LTs were checked at intervals until ALT was normal or approaching normal. Treatment was re-introduced using a staggered incremental dose increase regimen with omission of incriminated drugs and consequential extension of treatment.

We considered as “persistent” the cases in which liver enzymes remained elevated for more than four weeks despite discontinuation of therapy. For TB patients, this interval is crucial, because it corresponds to the time after which we must re-initiate the TB treatment in cases of suspension.

Severe and persistent elevation of liver transaminases and or hepatic cholestasis despite discontinuation of therapy, along with other suspected liver-related conditions, were the indications for liver biopsy (LB) in ten patients. 

The liver biopsy was performed under ultrasound or CT guidance (core not smaller than 1 cm, taken intercostally from the right lobe of the liver) under local analgesia with xylocaine. Written informed consent was obtained from all patients. Routine use of ultrasound was helpful in determining the best location for the biopsy. The area was prepped and draped in a sterile manner. The biopsy frustule was fixed in formalin and embedded in paraffin. LB patients underwent routine treatment for histopathological examination. Tissue sections were stained with hematoxylin and eosin, Masson’s trichrome, Perls’ stain, PAS and PAS-Diastase (PAS-D). CK7 immunostaining was performed to show ductular reaction and ductular metaplasia of hepatocytes. For molecular studies, DNA was selectively extracted with a QIAamp Tissue Kit (Qiagen GmbH, Hilden, Germany) from paraffin sections, and PCR detection of mycobacterium DNA was performed in all samples. Two experienced histopathologists evaluated the histological changes in the liver sections.

Data were collected from electronic medical records. Socio-demographic characteristics, including sex, age, ethnicity and smoking status, as well as clinical characteristics such as comorbidities and any ongoing therapy, were recorded. Descriptive analysis was conducted to characterize subjects enrolled in this study. Statistical analyses were conducted using StataCorp. 2023 (Stata Statistical Software: Release 13, StataCorp LP, College Station, TX, USA). 

## 3. Results

Ten patients who underwent LB in the period from January 2017 to December 2022 were extracted from the database and included in the retrospective study cohort. The main characteristics of the 10 patients are shown in Table 1. Most patients (70%) were male. The mean age of the patients was 38.3 (range 19–66) years. None of the patients were HIV-positive. Two patients tested positive for anti-HCV antibodies (viral load detectable in both patients). All patients had a form of pulmonary tuberculosis. One also had pleural localization and another hepatic (post-biopsy diagnosis). Seven patients (70%) had new cases of tuberculosis. Half of the ten patients (50%) had completely drug-sensitive tuberculosis, while five had rifampicin-resistant/multidrug-resistant (RR/MDR) tuberculosis [12]. The most common comorbidities were chronic hepatitis C (2/10) and cardiovascular disease (3/10). Half of the patients (5/10) had a history of alcohol abuse.

With regard to concomitant medications, two patients were on long-term antihypertensive drugs, one patient was on long-term hypoglycemic drugs, and one patient was on long-term oral anticoagulant (warfarin). None of the patients were going through immunosuppressive therapy. The number of drugs included in the ATT regimen in this cohort ranged from four to six (Table 1). The time interval from the onset of antituberculosis therapy to the detection of hepatotoxicity ranged from 30 to 330 days, with a mean time of 104 days. All patients had normal liver function before the diagnosis of TB-DILI. 

As for the severity of liver injury, eight patients had grades 1–3 DILI while only two patients had grades 4–5 DILI. In all patients, DILI required temporary suspension of the antituberculosis treatment. All patients were treated after onset of DILI according to local protocol and all completed treatment. The clinical manifestations of anti-TB DILI reported in our study population included nausea and loss of appetite (*n* = 10), fatigue (*n* = 10) and rash (*n* = 1). According to the clinical classification, eight patients were diagnosed with the hepatocellular type of DILI, one patient had the cholestasis type, and one patient reported a mixed-type injury. The laboratory analyses are shown in Table 2. 

**Figure 1:**
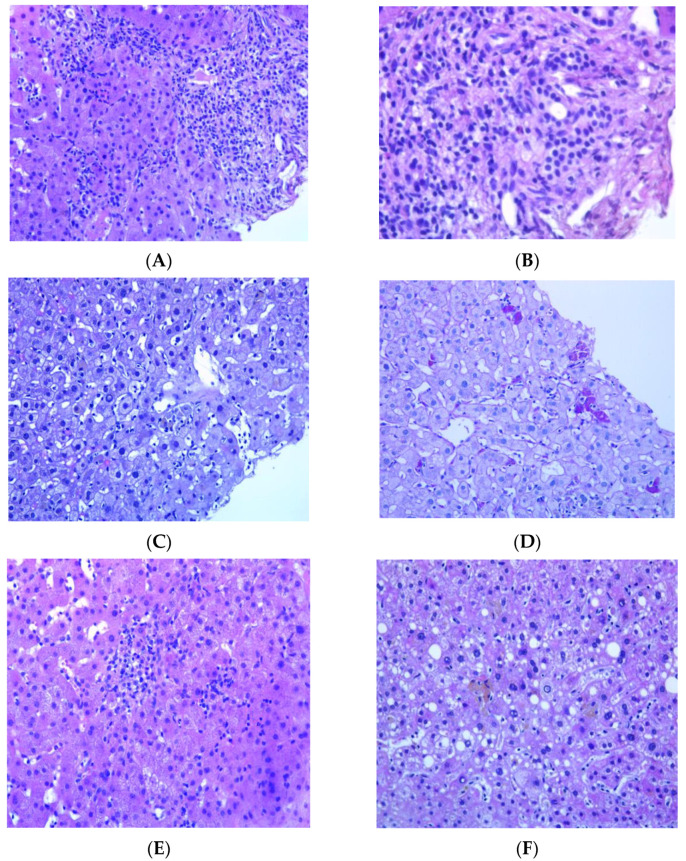
Histological features of DILI. (**A**) Interface hepatitis characterized by portal inflammatory infiltrate extending into the adjacent parenchyma; (**B**) higher magnification shows the presence of occasional eosinophils; (**C**,**D**) enlarged sinusoidal Kupffer cells (**C**) and clusters of macrophages revealed via histochemical staining with PAS-Diastase (**D**); (**E**) lobular confluent necrosis affecting groups of hepatocytes; and (**F**) micro- and macro-vesicular steatosis.

Histopathological diagnosis revealed liver damage due to DILI (Figure 1) in 5/10 (50%) cases (Table 2). The other cases were diagnosed as chronic viral hepatitis 2 (20%), along with one (10%) case of nonalcoholic fatty liver disease (NASH), one (10%) case of granulomatous hepatitis due to tuberculosis and one (10%) case of minimal and nonspecific hepatitis. Histological examination revealed a mild-to-moderate inflammatory infiltrate of the portal tract with lymphocytes (Figure 1A), macrophages with PAS D pigment mixed with some neutrophils and rare eosinophils (Figure 1B). Intralobular inflammation with confluent necrosis (Figure 1E) and aggregates of macrophages with positive PAS-D pigment (Figure 1C,D) were observed. One case showed large and small droplet steatosis affecting more than 5% of the liver parenchyma (Figure 1F). In one case, liver biopsy showed necrotizing granulomatous hepatitis suggestive of mycobacterial infection and confirmed to be mycobacterium tuberculosis via PCR testing. All other patients had negative results on liver samples. 

Table 2 shows the four anatomopathological criteria identified by the authors as specific to DILI (with a sensitivity of 91%) in the pathological description of the biopsies of our patients: portal inflammation, fibrosis, portal neutrophils and plasma cells and intracellular (hepatocellular) cholestasis. We found an association between the criteria and the diagnosis of DILI (mean value of five non-DILI patients 1.6; mean value of five patients with DILI 3.6; *p* = 0.0077, comparison of two means—Med calc).

## 4. Discussion

The present study describes the histopathological features of liver biopsies from ten tuberculosis patients who developed suspected DILI during treatment.

Effective treatment of tuberculosis requires a combination of bactericidal and/or bacteriostatic drugs. The combination of these drugs is regulated by the recommendations of the World Health Organization (WHO) [8,9]. All adverse events reduce the effectiveness of treatment, possibly contributing to treatment failure, relapse or the emergence of drug resistance [13].

During the study period, we diagnosed 1900 new cases of pulmonary and extrapulmonary tuberculosis at the INMI Spallanzani and started their treatment. We observed increased transaminases in 16% of patients. Symptoms and liver abnormalities usually resolved after stopping treatment. The rechallenge of drugs was progressive and chosen according to local institutional protocol.

Hepatic drug reactions can occur at any time during the treatment period, although DILI has been described to generally occur within 8 weeks of initiation [14]. In our study, 7 out of 10 patients (70%) developed hepatitis within 2 months (mean time 104 days; range 30–330), consistent with other published studies [15,16].

Well-known risk factors for TB-DILI are a low body mass index (BMI), metabolic isoniazid acetylator (INH) status, age, gender, metabolic factors, drug interactions and consumption of alcohol [17].

Although no patients reported alcohol consumption during treatment, it cannot be conclusively stated that, given the high percentage of referred history of alcohol intake (50%), concomitant unreferred use of alcohol did not influence the severity of the episode [18]. Five patients in this case series were RR/MDR patients. Risk factors of TB-DILI or ab-normal liver functioning include the type and dosage of drugs, the duration of medication and the combination of medications [19,20]. We previously reported that 8.1% of MDR TB patients experienced liver toxicity, demonstrating that the onset of drug-induced hepatitis is also a relevant event in the treatment of MDR/XDR TB [13].

There is no widely agreed-upon definition of TB DILI, and most definitions focus on the value of ALT. The DILI Expert Working Group has defined consensus threshold criteria valid for any drug-related DILI, including ALT- and ALP-based criteria [21]. In this context, the lack of standardized diagnostic criteria makes the diagnosis difficult even for experts, so liver biopsy could prove useful in selected cases. While liver function tests are commonly used to monitor liver function during ATT, liver biopsy can be a valuable tool to establish the differential diagnosis, assess the severity and guide the management of DILI [22].

Traditionally, histology has been considered the gold standard in the diagnosis of chronic liver disease. Furthermore, specific liver diseases, including DILI, have been diagnosed with the help of laboratory tests and, possibly, histological data of the liver. Based solely on laboratory tests such as ALT and ALP, DILI and other forms of liver injury are now classified as hepatocellular, cholestatic or mixed liver injury. However, there remain rare cases of diagnostic uncertainty regarding alternative causes in which liver biopsy should be considered as the final diagnostic approach, provided that the patient benefits from this procedure.

When faced with clinical suspicion of DILI, the indications for performing a biopsy could be the presence of multiple possible etiologies; information on the potential mechanism of damage; assessment of the severity of the injury to enable a clinical decision; unrecognized liver disease diagnosis; and exclusion of autoimmune hepatitis [23].

As regards TB-DILI, the criteria based on ALP may miss the diagnosis of hepatic localization of the disease, as we saw in the case of the patient with granulomatous hepatitis [24].

In our study population, we decided to perform liver biopsy in ten selected patients due to the persistence of the damage despite the suspension of drugs. Although the role of liver histology in the management of DILI and TB-DILI is still uncertain, we do believe that it has been helpful. After reviewing the liver histology, we could exclude DILI in 5/10 patients. 

In one patient, we found necrotizing granulomatous inflammation with positive ZN. Hepatic TB lacks typical clinical symptoms and imaging diagnosis so can easily be misdiagnosed, which may result in the interruption of treatment. In this case, a biopsy provided additional diagnostic data. Two patients (both RR/MDR) in this case series had chronic HCV infection (Table 1) [25]. Hepatitis C virus (HCV) chronic infection is more frequent in people affected by TB than in the general population, and it is a well-known concurrent risk factor for the development of TB-DILI [26].

The pharmaceutic armamentarium for MDR-TB is limited and includes drugs with potential liver toxicity. Reducing the cumulative risk of hepatic side effects in these patients by treating HCV chronic infection is crucial to completing a long and challenging therapeutic regimen. Recent data from a study by Tunesi and colleagues show that concomitant treatment for HCV and MDR/RR-TB was effective and well-tolerated with sustained virologic response and fewer common adverse events in MDR TB patients [27]. We observed this for two patients in this case series as well.

In one patient, the LB showed diffuse steatosis and mixed inflammatory infiltrate with foci of perivenular necrosis with hypertrophic Kupffer cells containing PASD+ macrophages, consistent with a diagnosis of nonalcoholic fatty liver disease (NAFLD). DILI in the context of NAFLD is a growing concern for practitioners. There are relatively few studies on the influence of NAFLD on the incidence of ATT-DILI. Many authors have reported a high incidence of ATT DILI in patients with pulmonary tuberculosis complicated by fatty liver [28] because ATT drugs can also exacerbate pre-existing NAFLD and accelerate the transformation from steatosis to steatohepatitis [29].

According to the Drug-Induced Liver Injury Network (DILIN), five distinct patterns of liver injury can be recognized: acute hepatitis, chronic hepatitis, acute cholestatic, chronic cholestatic and cholestatic hepatitis [7]. Gourishankar et al. reported moderate-to-severe and lobular lymphocyte and plasmocytic portal inflammation, prominent ductal proliferation, moderate cholestasis (predominantly hepatocellular and canalicular), hepatocellular damage and stage 3 bridging fibrosis in a young patient treated with isoniazid [30]. Consistent with these data, all five ATT-DILI patients in this case series exhibited hepatocellular injury [31].

A proportion of patients with drug-induced liver injury (DILI) present with autoantibodies, leading to the current concept of autoimmune-like DILI [32]. In our case series, we did not find any patients with this diagnosis.

Given the high diagnostic value reported for the patients described in this case series, we speculate that liver biopsy and histologic examination of the liver are likely to be beneficial in identifying specific liver diseases, as well as in ATT DILI. These can aid in confirming the diagnosis, evaluating disease severity and guiding treatment decisions. It is, however, essential to recognize that liver biopsy carries inherent risks, including bleeding and sampling variability. Therefore, its indication should be balanced with clinical judgment and consideration of individual patient factors. Noninvasive methods, such as imaging and biomarkers, continue to evolve and may provide complementary information, further reducing the need for liver biopsy in the future.

The results of this study should be considered in light of several limitations, since it was a retrospective, single-center case series report study; hence, it is not representative of the whole target population.

## 5. Conclusions

Severe and persistent elevations in hepatic transaminases and/or hepatic cholestasis despite discontinuation of therapy and suspicion of other liver damage conditions are indications for liver biopsy, which remains a valuable tool in the evaluation of selected patients with TB-DILI for many reasons. First, it provides more information about the state of the liver than any other single test. Secondly, the analysis of the type of lesion provides a pathological differential diagnosis that can be decisive for the differential diagnosis. Third, it could help establish a diagnosis of extrapulmonary tuberculosis. However, the decision to perform a liver biopsy should be based on clinical judgment, considering the benefits and risks of the procedure. Careful monitoring of liver function tests and timely recognition of liver damage can help prevent serious liver damage and improve patient prognosis. Further research is needed to develop noninvasive biomarkers for the diagnosis and management of ATT-induced liver injury.

## Figures and Tables

**Table 1:** Patients’ characteristics.

Pt. N.	Sex Age	Country of Birth	Comorbidities	Alcohol	HBV/HCV Infection	Diagnosis	Microbiology	Radiology	Treatment	Total Drugs	TD
1	F 41	Romania	uterine cancer	no	HCV	new	AFB+/culture-positive	Pulm; cavitation	Lfx, Cs, Etho, PAS, Lzd	6	30
2	M 66	Italy	hypertension	yes	HCV	new	AFB+/culture-positive	Pulm; cavitation	E, Z, S, Lfx, Lzd	6	330
3	M 27	Ukraine	none	no	neg	new	AFB-/culture-positive	Pulm; cavitation	E, Mfx, Amk, Cs	4	240
4	M 56	Egypt	diabetes	yes	neg	new	AFB+/culture-positive	Pulm; cavitation	R, H, Z, E	5	40
5	M 19	Cameron	none	no	neg	relapse	AFB+/culture-positive	Pulm; cavitation	R + H + E + Z	4	60
6	F 23	Egypt	none	no	neg	relapse	AFB+/culture-positive	Pulm; cavitation	R, H, Z, E	4	120
7	M 36	Eritrea	none	yes	neg	relapse	AFB+/culture-positive	Pulm + EPTB; cavitation	Lzd, Cfz, Cs, E, Amk, Bdq	5	65
8	F 42	Philippines	valvular replacement, chronic atrial fibrillation	no	neg	new	AFB+/culture-positive	Pulm; cavitation	Bdq, Mfx, Amk, Cfz, Cs, Lzd	6	60
9	M 37	Philippines	none	yes	neg	new	AFB-/culture-positive	EPTB	H, E, Lfx, Amk	4	30
10	M 36	Romania	none	yes	neg	new	AFB+/culture-positive	Pulm + EPTB; cavitation	R, H, Z, E	4	35

Legends: HBV = hepatitis B virus; HCV = hepatitis B virus; AFB = acid-fast bacilli; Lfx = levofloxacin; Cs = cycloserine; PAS = para-aminosalicylic acid; Lzd = linezolid; E = ethambutol; Amk = amikacin; H = isoniazid; Z = pyrazinamide; Cfz = clofazimine; Bdq = bedaquiline; R = rifampicin; Pulm: pulmonary TB; EPTB: extrapulmonary TB; TD: total days of exposure before AST/ALT elevation.

**Table 2:** Suspected TB DILI characteristics.

Pat. N	Symptoms	Jaundice	Management AE	AST U/L	ALT U/L	ALP U/L	Severity Grading WHO	RUCAM Value	Pattern	DILI Causality Assessment	Hystology Findings	4 DILI-Favoring Features	Diagnosis
1	fatigue, nausea, lost of appetite	no	suspension	316	749	156	4	12.5	hepatocellular	3, possible	Mild necroinflammatory activity, and portal fibrosis (Ishak Grade 5 and Stage 2)	2	Chronic viral hepatitis HCV correlated
2	fatigue, nausea, lost of appetite	no	suspension	287	403	271	3	8.4	hepatocellular	4, possible	Portal tracts enlarged due to edema and fibrosis and containing inflammatory infiltrate of mild/moderate density, consisting mainly of lymphocytes, sometimes aggregated to form follicles, more than occasional plasma cells and some PASD+ macrophages. This inflammatory infiltrate sometimes surrounds the native bile duct with phenomena of lymphocytic cholangitis. The interlobular bile ducts demonstrate features of cholangiocyte senescence. In the acinar site there is an inflammatory intracinar lymphomonocytic infiltrate with spotty necrosis, some apoptotic bodies and hypertrophy of Küppfer cells with PASD+ pigment. Some areas appear regenerative in appearance. (Ishak Grade 9 Stage 4).	3	Chronic viral hepatitis, HCV correlated
3	fatigue, nausea, lost of appetite	no	suspension	137	407	139	3	12.5	hepatocellular	1, unlikely	Portal tracts containing minimal inflammatory lymphocytic infiltrate with some PASD+ macrophages and occasional neutrophilic granulocytes. Interlobular bile ducts always visible. Diffuse steatosis with large and medium-sized vesicles is observed in the parenchymal area, greater than 66%. There is a mixed inflammatory infiltrate with foci of mediational and perivenular necrosis (spotty) and some lipogranulomas. Küppfer cells appear hypertrophic and contain PASD+ pigment. Iron deposit of the type with weak and diffuse staining (ferritin) of the type and of the small granule type at the level of the hepatocytes in zones 1 and 2 and of the non-confluent granule type at the level of the Küppfer cells. Minimal increase in collagen density in some portal tracts.	0	Non-alcoholic steatohepatitis (nash). Secondary iron overload
4	fatigue, nausea, lost of appetite	no	suspension	440	426	152	3	8.4	hepatocellular	8, probable	inflammatory infiltrate consisting mainly of lymphocytes with some plasma cells neutrophils granulocytes and PASD+ macrophages. In the lobular area, the inflammatory lymphomonocytic infiltrate is moderate with spotty necrosis, sometimes confluent; in the lumen of the sinusoids the resident macrophages are hypertrophic/hyperplastic and several stellate cells (HSC) are also evident.	4	TB-DILI (acute)
5	fatigue, nausea, lost of appetite	yes	suspension	2134	1180	542	4	11.6	hepatocellular	4, possible	Inflammatory infiltrate of mild to moderate density consisting predominantly of lymphocytes that shows no propensity to pass the lamina. In the parenchymal site various aspects of hepatocellular suffering are observed associated with pictures of dysarray of the acinus and lymphohistiocytic infiltration with the participation of neutrophils. Sinusoids reduced in amplitude also due to the presence of numerous macrophages. Mono- and polymorphonuclear inflammatory cells are dispersed in the sinusoids.	3	TB DILI (acute)
6	fatigue, nausea, lost of appetite	no	suspension	472	480	161	3	8.2	hepatocellular	4, possible	Portal spaces containing mild inflammatory infiltrate consisting of lymphocytes, PASD+ macrophages, and occasional eosinophilic granulocytes. Minimal and focal involvement of the portoparenchymal limiting plate. The bile ducts are visible in most of the portal tracts, and rarely any lymphocytes permeate the basement membrane. At the periportal level, some intermediate immunophenotype cells are observed. The ductular reaction is absent. Spotty necrosis foci are observed in the parenchyma with a prevalence of macrophages with PASD+ pigment, often grouped in clusters. Minimal portal fibrosis.	4	TB-DILI (partial resolution)
7	fatigue, nausea, lost of appetite	no	suspension	256	330	321	3	14.1	hepatocellular	5, possible	mild macrovesicular steatosis, focal microvesicular steatosis. With the aid of histochemical stains for the reticulum, PAS-D and Perls Iron staining, we also note: dilatation of the sinusoids with Küpffer cell hyperplasia with some inflammatory cells represented by lymphocytes and clusters of phagocytosing macrophages pigment partly referable to ceroid (PAS-D+) and partly refractive and slightly bluish with iron staining (Perls) as for hemosiderin. Mild biliary phenotype of perivenular and periportal hepatocytes is observed on immunohistochemical staining for cytokeratin 19. Mild perivenular and sinusoidal fibrosis	3	TB-DILI (partial resolution)
8	fatigue, nausea, lost of appetite	no	suspension	189	220	115	3	4.6	mixed	5, possible	needle bioptic fragments of liver with preserved structure characterized by mild inflammatory infiltrate of the portal spaces consisting of rare lymphocytes and rare macrophages with PAS-D+ pigment, minimal macrovacuolar steatosis (<5% of the liver parenchyma). Rare acidophilic bodies, focus of spotty necrosis. Rare glycogenated nuclei in the lobule. No iron deposits are observed. Fibrous expansion of some portal spaces (F0-F1 sec Metavir)	2	minimal non-specific hepatitis
9	fatigue, nausea, lost of appetite	no	suspension	152	270	469	3	1.2	cholestatic	8, probable	necrotizing granulomatous inflammation. A single bacillary formation ZN+ is found, which can be referred to an acid-fast bacillus	1	TB granulomatous hepatitis
10	fatigue, nausea, lost of appetite	no	suspension	98	298	100	3	16.9	hepatocellular	4, possible	mild and focal portal inflammatory infiltrate consisting of neutrophil granulocytes, eosinophils, PASD+ macrophages and rare lymphocytes. ductular proliferation spotty necrosis, hepatocyte rosettes	4	TB-DILI

## Data Availability

All relevant data are within the manuscript. Raw data are accessible, if requested, from National Institute for Infectious Diseases “L. Spallanzani” Library to E-mail address: biblioteca@inmi.it.

## References

[1] (2022). Global Tuberculosis Report. https://www.who.int/publications/i/item/9789240061729.

[2] Rathi C., Pipaliya N., Patel R., Ingle M., Phadke A., Sawant P. (2017). Drug Induced Liver Injury at a Tertiary Hospital in India: Etiology, Clinical Features and Predictors of Mortality. Ann. Hepatol..

[3] Abbara A., Chitty S., Roe J.K., Ghani R., Collin S.M., Ritchie A., Kon O.M., Dzvova J., Davidson H., Edwards T.E. (2017). Drug-induced liver injury from antituberculous treatment: A retrospective study from a large TB centre in the UK. BMC Infect. Dis..

[4] Zhong T., Fan Y., Dong X.L., Guo X., Wong K.H., Wong W.T., He D., Liu S. (2021). An Investigation of the Risk Factors Associated with Anti-Tuberculosis Drug-Induced Liver Injury or Abnormal Liver Functioning in 757 Patients with Pulmonary Tuberculosis. Front. Pharmacol..

[5] Brennan P.N., Cartlidge P., Manship T., Dillon J.F. (2021). Guideline review: EASL clinical practice guidelines: Drug-induced liver injury (DILI). Frontline Gastroenterol..

[6] Danan G., Benichou C. (1993). Causality assessment of adverse reactions to drugs--I. A novel method based on the conclusions of international consensus meetings: Application to drug-induced liver injuries. J. Clin. Epidemiol..

[7] Kleiner D.E. (2009). The pathology of drug-induced liver injury. Semin. Liver Dis..

[8] WHO Consolidated Guidelines on Tuberculosis: Module 4: Treatment: Drug-Susceptible Tuberculosis Treatment. https://www.who.int/publications/i/item/9789240048126.

[9] WHO Consolidated Guidelines on Tuberculosis: Module 4: Treatment: Drug-Resistant Tuberculosis Treatment. https://www.who.int/publications/i/item/9789240007048.

[10] World Health Organization (WHO) (2015). Active Tuberculosis Drugsafety Monitoring and Management (aDSM). Framework for Implementation. (WHO/HTM/TB/2015.28). Geneva, WHO. http://apps.who.int/iris/bitstream/10665/204465/1/WHO_HTM_TB_2015.28_eng.pdf.

[11] Saukkonen J.J., Cohn D.L., Jasmer R.M., Schenker S., Jereb J.A., Nolan C.M., Peloquin C.A., Gordin F.M., Nunes D., Strader D.B. (2006). ATS (American Thoracic Society) Hepatotoxicity of Antituberculosis Therapy Subcommittee. An official ATS statement: Hepatotoxicity of antituberculosis therapy. Am. J. Respir. Crit. Care Med..

[12] Meeting Report of the WHO Expert Consultation on Drug-Resistant Tuberculosis Treatment Outcome Definitions. https://www.who.int/publications/i/item/9789240022195.

[13] Gualano G., Mencarini P., Musso M., Mosti S., Santangelo L., Murachelli S., Cannas A., Di Caro A., Navarra A., Goletti D. (2019). Putting in harm to cure: Drug related adverse events do not affect outcome of patients receiving treatment for multidrug-resistant Tuberculosis. Experience from a tertiary hospital in Italy. PLoS ONE.

[14] Hosford J.D., von Fricken M.E., Lauzardo M., Chang M., Dai Y., Lyon J.A., Shuster J., Fennelly K.P. (2015). Hepatotoxicity from antituberculous therapy in the elderly: A systematic review. Tuberculosis.

[15] Gülbay B.E., Gürkan O.U., Yildiz O.A., Onen Z.P., Erkekol F.O., Baççioğlu A., Acican T. (2006). Side effects due to primary antituberculosis drugs during the initial phase of therapy in 1149 hospitalized patients for tuberculosis. Respir. Med..

[16] Gaude G.S., Chaudhury A., Hattiholi J. (2015). Drug-induced hepatitis and the risk factors for liver injury in pulmonary tuberculosis patients. J. Fam. Med. Prim. Care..

[17] Tostmann A., Boeree M.J., Aarnoutse R.E., de Lange W.C., van der Ven A.J., Dekhuijzen R. (2008). Antituberculosis drug-induced hepatotoxicity: Concise up-to-date review. J. Gastroenterol. Hepatol..

[18] Ramappa V., Aithal G.P. (2013). Hepatotoxicity Related to Anti-tuberculosis Drugs: Mechanisms and Management. J. Clin. Exp. Hepatol..

[19] Lee S.S., Lee C.M., Kim T.H., Kim J.J., Lee J.M., Kim H.J., Ha C.Y., Kim H.J., Jung W.T., Lee O.J. (2016). Frequency and risk factors of drug-induced liver injury during treatment of multidrug-resistant tuberculosis. Int. J. Tuberc. Lung Dis..

[20] Aithal G.P., Watkins P.B., Andrade R.J., Larrey D., Molokhia M., Takikawa H., Hunt C.M., Wilke R.A., Avigan M., Kaplowitz N. (2011). Case definition and phenotype standardization in drug-induced liver injury. Clin. Pharmacol. Ther..

[21] Freitas M., Magalhães J., Marinho C., Cotter J. (2020). Looking beyond appearances: When liver biopsy is the key for hepatic tuberculosis diagnosis. BMJ Case Rep..

[22] Clinton J.W., Kiparizoska S., Aggarwal S., Woo S., Davis W., Lewis J.H. (2021). Drug-Induced Liver Injury: Highlights and Controversies in the Recent Literature. Drug Saf..

[23] Costa-Moreira P., Gaspar R., Pereira P., Lopes S., Canão P., Lopes J., Carneiro F., Macedo G. (2020). Role of liver biopsy in the era of clinical prediction scores for "drug-induced liver injury" (DILI): Experience of a tertiary referral hospital. Virchows Arch..

[24] Wu Z., Wang W.L., Zhu Y., Cheng J.W., Dong J., Li M.X., Yu L., Lv Y., Wang B. (2013). Diagnosis and treatment of hepatic tuberculosis: Report of five cases and review of literature. Int. J. Clin. Exp. Med..

[25] Ungo J.R., Jones D., Ashkin D., Hollender E.S., Bernstein D., Albanese A.P., Pitchenik A.E. (1998). Antituberculosis drug-induced hepatotoxicity. The role of hepatitis C virus and the human immunodeficiency virus. Am. J. Respir. Crit. Care Med..

[26] Musso M., Mosti S., Gualano G., Mencarini P., Urso R., Ghirga P., Rianda A., Del Nonno F., Goletti D., Palmieri F. (2019). Hepatitis C virus infection: A challenge in the complex management of two cases of multidrug-resistant tuberculosis. BMC Infect. Dis..

[27] Tunesi S., Dû D.L., Gualano G., Millet J.P., Skrahin A., Bothamley G., Casas X., Goletti D., Lange C., Musso M. (2022). TBnet, The ESGMYC, and The French MDR-TB Group. Co-administration of treatment for rifampicin-resistant TB and chronic HCV infection: A TBnet and ESGMYC study. J. Infect..

[28] Zhao H., Wang Y., Zhang T., Wang Q., Xie W. (2020). Drug-Induced Liver Injury from Anti-Tuberculosis Treatment: A Retrospective Cohort Study. Med. Sci. Monit..

[29] Jiang F., Yan H., Liang L., Du J., Jin S., Yang S., Wang H., Hu T., Zhu Y., Wang G. (2021). Incidence and risk factors of anti-tuberculosis drug induced liver injury (DILI): Large cohort study involving 4652 Chinese adult tuberculosis patients. Liver Int..

[30] Gourishankar A., Navarro F., Debroy A.N., Smith K.C. (2014). Isoniazid hepatotoxicity with clinical and histopathology correlate. Ann. Clin. Lab. Sci..

[31] Neuberger J., Cain O. (2021). The Need for Alternatives to Liver Biopsies: Non-Invasive Analytics and Diagnostics. Hepat. Med..

[32] Weber S., Benesic A., Rotter I., Gerbes A.L. (2019). Early ALT response to corticosteroid treatment distinguishes idiosyncratic drug-induced liver injury from autoimmune hepatitis. Liver Int..

